# Electromyography Signals in Embedded Systems: A Review of Processing and Classification Techniques

**DOI:** 10.3390/biomimetics10030166

**Published:** 2025-03-10

**Authors:** José Félix Castruita-López, Marcos Aviles, Diana C. Toledo-Pérez, Idalberto Macías-Socarrás, Juvenal Rodríguez-Reséndiz

**Affiliations:** 1 Facultad de Ingeniería, Universidad Autónoma de Querétaro, Santiago de Querétaro 76240, Mexico; jcastruita04@alumnos.uaq.mx (J.F.C.-L.); diana.toledo@uaq.mx (D.C.T.-P.); 2 Facultad de Ciencias Agrarias, Universidad Estatal Península de Santa Elena (UPSE), Santa Elena 240204, Ecuador; imacias@upse.edu.ec

**Keywords:** embedded systems, EMG, artificial intelligence, classification algorithms, FPGA, SoC

## Abstract

This article provides an overview of the implementation of electromyography (EMG) signal classification algorithms in various embedded system architectures. They address the specifications used for implementation in different devices, such as the number of movements and the type of classification method. Architectures analyzed include microcontrollers, DSP, FPGA, SoC, and neuromorphic computers/chips in terms of precision, processing time, energy consumption, and cost. This analysis highlights the capabilities of each technology for real-time wearable applications such as smart prosthetics and gesture control devices, as well as the importance of local inference in artificial intelligence models to minimize execution times and resource consumption. The results show that the choice of device depends on the required system specifications, the robustness of the model, the number of movements to be classified, and the limits of knowledge concerning design and budget. This work provides a reference for selecting technologies for developing embedded biomedical solutions based on EMG.

## 1. Introduction

Recently, multiple studies on the use of bioelectric signals from the human body have aimed to develop new technologies for their analysis and interpretation and thus achieve the development or optimization of processes or models [1]. One of the most important types of biosignals is electromyography (EMG) signals, which have been extensively studied as they provide data for analyzing muscle activity. An EMG signal is a biopotential representing the electrical currents generated during muscle contraction and relaxation. However, since these signals originate from muscle activity controlled by the nervous system, they are very complex in their raw representation [2].

EMG signals are a fundamental tool in the development of biomimetic technologies, whose purpose is to replicate the natural behavior of human body movements [3]. In this context, an EMG gesture is defined as a sequence of voluntary muscle activations resulting in distinguishable movements captured in EMG signals [4]. The analysis of these signals enables the interpretation of different gestures, which is crucial for applications such as intelligent prosthetics, exoskeletons, and control systems based on human–machine interfaces.

Due to their complexity, classifying EMG signals is crucial to correctly interpreting the information and obtaining useful data. To achieve this, certain features representing the signal need to be extracted, and artificial intelligence algorithms are usually used for classification. Among the most prevalent techniques are supervised learning algorithms such as support vector machine (SVM) and k-nearest neighbor (KNN) algorithms. Other techniques that have recently gained popularity are neural networks, such as multilayer perceptron networks (MLPs), which are among the most common. Convolutional neural networks (CNNs) are frequently used, as shown in [5]. Recently, classifiers with recurrent neural networks (RNNs) have been used, as the authors of [6] show. These and other artificial intelligence methods aim to achieve adequate performance in motion detection. However, achieving such performance comes at a high computational cost due to the model’s training process and hyperparameters. These external parameters define important aspects such as the model’s architecture and learning rate [2]. Therefore, implementing these methods in responsive applications, such as real-time systems, often requires powerful and costly computing equipment. In addition, these systems are difficult to transport due to their size and power requirements [1,7].

This situation raises a challenge when integrating EMG signal classification into wearable systems. In this context, accurate and efficient classification of EMG signals on embedded devices has gained significant importance, especially for real-time and field applications such as smart prostheses, rehabilitation devices, and gesture-based control systems. Thus, for extracting features and classifying these signals, it is fundamental to find technologies that allow an efficient implementation in terms of time and energy consumption. Some devices and processors have already been used for the above applications. For example, microcontrollers are used for feature extraction and AI model inference for classification in [8,9]. In [10], digital signal processors (DSPs) or field-programmable gate arrays (FPGAs) are used. In [11], feature extraction and classification are performed on System-on-Chip (SoC) devices such as Raspberry Pi^®^, which the UK-based Raspberry Pi Foundation manufacture, or Jetson^®^ GPUs, which is manufactured by NVIDIA, an American company based in Santa Clara, California, USA. Recently, neuromorphic systems have been used for these applications, as shown in [12].

This work aims to review studies on implementing embedded devices for processing and classifying EMG signals and the techniques used. It analyzes the processing times and energy consumption reported in these studies. The focus is on a detailed analysis of device implementations such as microcontrollers, DSPs, FPGAs, SoCs, and neuromorphic chips. In terms of processing, the study is primarily concerned with extracting features representing the EMG signal and shows which features are most commonly implemented and on which devices. The precision metrics obtained in the studies are presented in terms of classification, and the preferred classification models for each type of embedded architecture are analyzed. This article provides an overview of embedded technologies used to analyze EMG signals for motion discrimination, with potential applications in portable devices that can be integrated into daily life.

The most important contributions of the work are as follows:

- Novel approach: few studies take this analytical perspective and emphasize using embedded systems to implement artificial intelligence algorithms in classifying EMG signals.
- State-of-the-art vision: This work provides a comprehensive overview of the state of the art in embedding EMG classification models for potential use in portable applications, such as smart prostheses. It serves as a reference for selecting the appropriate device for future studies.
- Identification of challenges and opportunities: The study identifies the challenges associated with current technologies and areas of opportunity for research, such as improving portability and reducing costs.

This paper is structured as follows: Section 2 contains the methodology for article selection. Section 3 gives an overview of the features used to identify gestures with different embedded devices. Section 4 gives an overview of works in which embedded devices were used for classification and presents the results obtained and the study’s implications. Section 5 contains a discussion and comparison of the use of different devices. Finally, the study’s conclusions are presented in Section 6.

## 2. Methodology

This section describes the methodology for article selection and exclusion. To conduct this review, the Preferred Reporting Items for Systematic Reviews and Meta-Analysis (PRISMA) guidelines were used [13]. An extensive search for indexed articles was conducted in scientific databases such as Scopus, IEEE Xplore, PubMed, and Google Scholar. Publications from 2010 to 2025 were considered, with some exceptions for particularly relevant studies.

Keywords such as “EMG signals”, “embedded systems”, “classification”, “neuromorphic systems”, “FPGA”, “SoC”, “GPU”, “Raspberry”, “DSP”, “microcontroller”, and “artificial intelligence”, as well as combinations of these, were used. Initially, the articles were screened by reviewing their titles and abstracts to determine their inclusion. Duplicate articles were discarded. Subsequently, the full texts were reviewed to assess the relevance of the content and finalize the selection. The following criteria were applied for article selection:

- Feature extraction of EMG signals on embedded devices.
- Classification of EMG signals on embedded devices.
- Works that did not describe the architecture used in detail were excluded.
- Works in which the embedded device was only used to capture the EMG signal were excluded.

**Figure 1:**
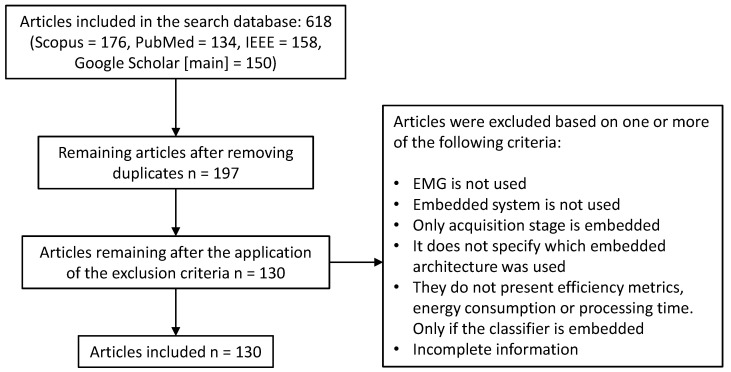
General methodology for article selection.

The results were recorded in tables summarizing the key features of each study. A total of 130 articles were analyzed in this work. The article selection process is illustrated in Figure 1.

## 3. EMG Signals

EMG signals provide information about muscle strength, movement, and fatigue. This muscle activity is recorded using electrodes that are either invasive (e.g., with needles inserted into the muscle) or non-invasive (on the skin) [7,14]. The EMG signal represents the action potentials of the muscle fibers [7].

### Feature Extraction in Embedded Devices

When EMG signals are used to record the electrical activity in the muscles, they contain information about the timing and intensity of movements [7]. The processing of these signals includes preprocessing for analysis and subsequent classification [2]. Feature extraction is a crucial step in this preprocessing, where metrics are derived from the acquired EMG signal samples. Typically, features are extracted in the time and frequency domain, although some techniques utilize both domains [14]. Features that provide high-motion discrimination should be selected. According to the literature, a classification accuracy exceeding 90% is considered acceptable, as in [15], where 93% was achieved, or in [16], where 90% was reached. However, this accuracy metric varies depending on the application and methods. Another important factor to consider when selecting features is that they should not require excessive computational resources to calculate, ensuring their potential use in embedded devices [17]. Table 1 lists the features used in the studies analyzed in this review and shows the domain in which each feature is located.

In Table 2, a compilation of works performing feature extraction on embedded devices is presented (Table A1).

**Figure 2:**
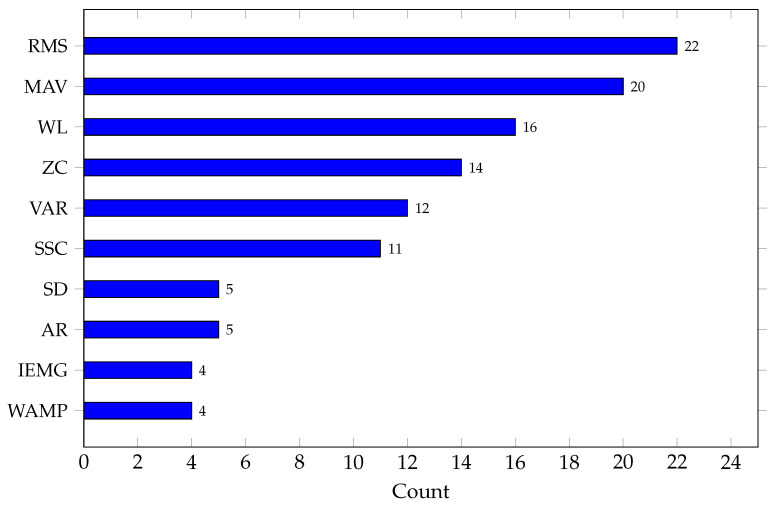
Top 10 features used in the reviewed studies.

According to the studies analyzed, the preferred features belong to the time domain, with RMS, MAV, WL, ZC, and VAR standing out. Figure 2 shows the top 10 features used in the studies analyzed, highlighting that they all belong to the time domain.

## 4. EMG Signal Classification in Embedded Devices

The classification of EMG signals is an important task in biomedical engineering. Artificial intelligence algorithms are often used for this purpose, as they have proven effective in identifying and distinguishing patterns in this type of signal. Table 3 lists the acronyms for the classification algorithms used in the studies examined.

A high level of computation is usually required to implement these algorithms, which is why computing systems that consume much power and, in some cases, cooling systems are used to maintain their operation, as is the case with servers and high-performance workstations [7,21].

The need for these large and energy-intensive systems is a limitation for applying these solutions in everyday life. This applies to real-time and field applications like intelligent prosthetics or gesture-controlled systems. For this reason, the trend is towards more efficient and compact alternatives, such as embedded devices and specialized hardware platforms [17]. A good practice in embedded systems, which has yielded positive results in processing times and classification accuracy, is to perform only model inference [63]. Training, on the other hand, is usually the most computationally intensive phase.

### 4.1. Inference of Algorithms

Algorithm inference on embedded devices, also known as edge inference or edge computing, refers to the process of running pre-trained artificial intelligence models on portable devices such as microcontrollers, FPGAs, SoCs, and other embedded devices [64,65]. Using inference eliminates the reliance on remote servers or the cloud, which was previously necessary for systems with higher processing capacity and larger size to perform operations [66]. This means that the logic and arithmetic of the algorithms are executed directly on the device into which the data are input.

**Figure 3:**
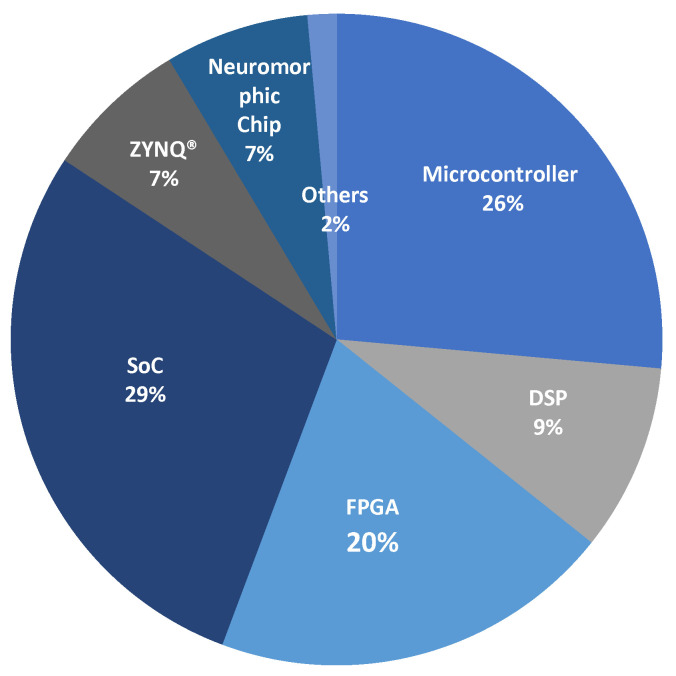
Most-used platforms in the inference of EMG classifiers according to the reviewed literature.

Several platforms stand out in terms of model inference for the classification of EMG signals. Figure 3 shows the percentage of use of these platforms based on the literature reviewed. SoCs lead with 29% of implementations due to their ease of executing artificial intelligence algorithms, thanks to programming capabilities in languages like Python. Microcontrollers, with 26% of implementations, follow them as they are the oldest technology on the market. In contrast, neuromorphic chips and ZYNQ devices were the architectures with the fewest model inference studies, at only 7%. The other platforms have similar percentages. In practice, there is no significant difference in preference for a particular technology, and all aim to enable portable real-time systems.

The following subsections report on the reviewed works that use different architectures for interpreting and discriminating EMG signals in embedded systems.

### 4.2. Microcontroller

Microcontrollers are an option for classifying EMG signals due to their low cost and good energy efficiency [9]. Although these devices have limited memory and processing power compared to an FPGA or DSP, they can still run classification algorithms and process signals using optimization techniques and less computationally intensive algorithms. In [9,11,19], LDA is used to classify signals as a lightweight algorithm compared to other techniques.

Table 4 shows some relevant studies that use microcontrollers to process EMG signals and give the accuracy percentage in motion prediction.

The methods used for signal classification via microcontrollers highlight the use of MLP, SVM, and LDA algorithms. These algorithms are less demanding in processing power, making them an excellent choice for using microcontrollers.

Microcontrollers are a suitable option for energy consumption when low power consumption is required. In [9], for example, an energy consumption of 29.7 mW was measured during the execution of an SVM classifier. In terms of processing time, microcontrollers also show reasonable performance for applications that do not require decision speeds below 100 ms.

**Figure 4:**
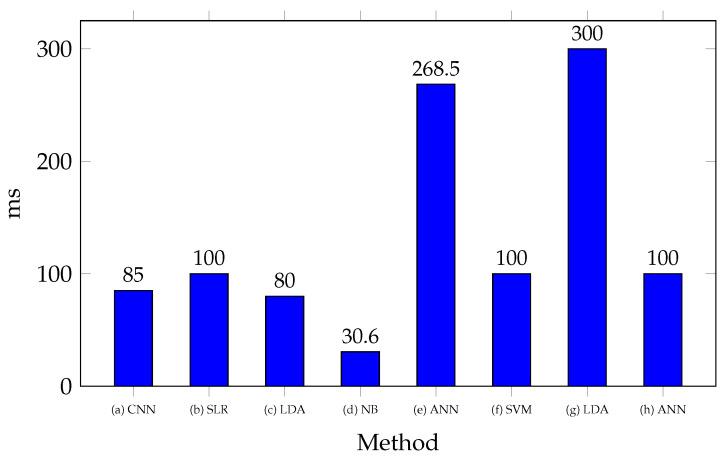
Processing times for different EMG classification techniques implemented on microcontrollers. References: (a) [70], (b) [38], (c) [74], (d) [42], (e) [47], (f) [77], (g) [55], (h) [78].

Figure 4 shows the processing times reported for classifying a gesture with microcontrollers. Variations are highlighted based on algorithm complexity. In [42], the shortest processing time is reported using the NB method, with a time of 30.6 ms, making it a strong candidate for applications requiring high processing speed. Meanwhile, in [74], 80 ms is reported using LDA, in [70], 85 ms with CNN, in [78], 100 ms with ANN, and in [77], 100 ms with SVM. These represent moderate processing times due to the use of more robust algorithms. In contrast, in [47], ANN was used with a processing time of 268.5 ms, while in [55], LDA was used with a processing time of 300 ms. These require significantly higher processing times, suggesting that more complex models may suffer from increased latency in real-time applications on microcontrollers. It is worth noting that in [55,74], the LDA method was employed, but with a significant difference in processing times. This can be primarily attributed to the fact that in [55], a higher number of channels were processed. Additionally, differences in implementation techniques or microcontroller architecture variations may have influenced the results. Similarly, in [47,78], ANN models were implemented, showing a considerable difference in processing times, mainly due to the specific neural network architecture used in each study.

These results indicate that lightweight models are preferable for microcontroller implementation, while complex models may require more powerful hardware to minimize latency.

### 4.3. Digital Signal Processor

DSPs are designed to process real-time signals and perform filtering, analysis, and transformation operations [33]. They are more efficient than general-purpose processors, although they are more expensive. DSPs are a choice for processing EMG signals due to their precision and speed. In addition, they are reconfigurable devices, so their performance can be optimized according to the specific requirements of each application. Table 5 shows relevant studies that use DSPs to classify EMG signals.

Regarding power consumption, [31] reported a power consumption of 40.3 mW for extracting four features in the time domain and 26.6 mW for performing classification with LDA. The same study documented that the device required 75 ms to complete the task from feature extraction to classification of a signal pattern. In contrast, in [33], it was reported that the device took between 200 and 300 ms to complete the task under the same conditions with eight movements and six sensors for detection, also using LDA for classification.

### 4.4. Field Programmable Gate Array

FPGAs are reconfigurable devices that enable the parallel implementation of algorithms as they are programmed at the hardware or gate level. This feature is advantageous for applications that require high processing speed [84]. These devices are suitable for classifying EMG signals due to their ability to process large amounts of real-time data and flexibility in implementing different classification algorithms. Due to their parallel programming structure, FPGAs are well suited for implementing neural networks, which are also based on a parallel architecture [85]. Table 6 shows some studies using FPGAs to classify EMG signals.

In [86], an FPGA was used to extract muscle synergies, which were then used in motion classification, achieving high precision in motion discrimination and low execution time. On the other hand, in [20], an SVM algorithm was derived on a Kintex 7^®^ FPGA for the classification of EMG signal, which showed significant improvements in execution time compared to the software-implemented model.

**Figure 5:**
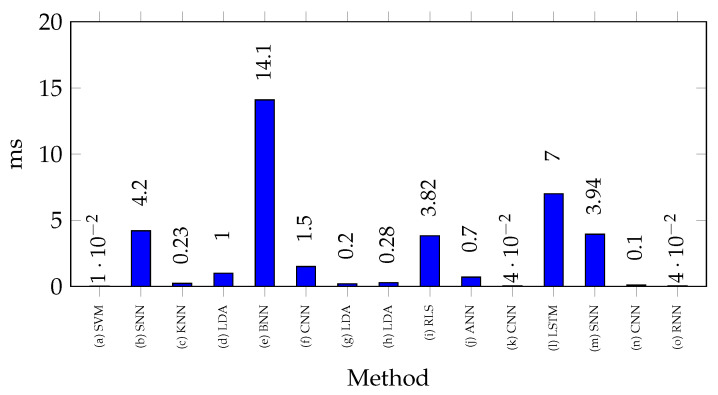
Processing times for different EMG classification techniques implemented on FPGA. References: (a) [86], (b) [1], (c) [10], (d) [26], (e) [87], (f) [35], (g) [40] (h) [41], (i) [91], (j) [94], (k) [98], (l) [101], (m) [88], (n) [104], (o) [104].

In the reported studies, the processing time for classification using FPGAs stands out as a design specifically tailored to the task of significantly optimizing the latency in model execution. Figure 5 shows the processing times recorded by the authors when performing inference on FPGAs. The figure indicates that most studies report processing times of less than 1 ms, which underlines the efficiency of FPGAs for the inference of classification models in real-time. Higher processing times for some classifiers may indicate higher computational complexity or a less optimized hardware design [26]. The highest reported processing time is found in [87], a time of 14.1 ms due to the model used, as it uses a binarized neural network approach. This approach is new and has the potential for further optimization. Other studies, such as [1,88], reported processing times of around 4 ms using spiking neural network (SNN) models. Since these models belong to the neural network category, they require longer processing times compared to other classifiers, such as the research of [86], which reported 0.01 ms using SVM, or [10], which recorded 0.23 ms with KNN.

Overall, using FPGAs for optimized EMG classification leveraging parallelism enables significantly faster processing times than architectures like microcontrollers or SoCs, making them well suited for real-time applications.

Regarding the energy efficiency of FPGAs, power consumption data usually refer to FPGA development boards and not to the chip alone. For example, in [86], a power consumption of 3.1 W was reported for running a classifier based on non-negative matrix factorization on a Pynq-Z1 board. Another reported power consumption was 3.8 W when running an SVM classifier on a Zynq-7000 board, as shown in [18].

### 4.5. System on a Chip

SoCs integrate multiple system components into a single chip, including CPU, GPU, memory, and communication interfaces [106]. Platforms such as the Raspberry Pi^®^ and Jetson Nano^®^ are widely used in signal processing because they can run complex algorithms in real-time with moderate power consumption. For example, the Raspberry Pi 3^®^ requires 4 W of power to operate, and a Jetson Nano^®^ was reported in [107] to consume 3.005 W when running a CNN model. These characteristics make SoCs suitable for classifying EMG signals, as they enable the efficient implementation of powerful machine learning algorithms, such as CNN, CRNN, and RVFLN. All this is achieved in compact and portable environments. Table 7 shows several studies using SoCs to classify EMG signals.

**Figure 6:**
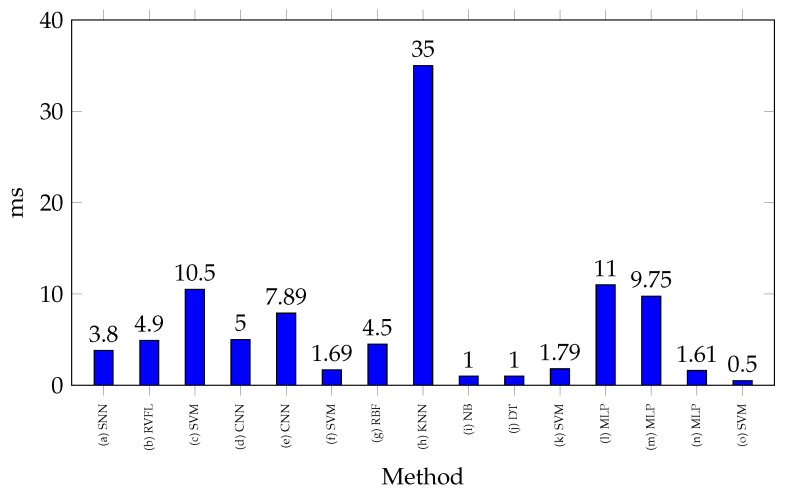
Processing times for different EMG classification techniques implemented on SoCs. References: (a) [1], (b) [29], (c) [29], (d) [107], (e) [111], (f) [111], (g) [17], (h) [32], (i) [36] (j) [36], (k) [36], (l) [37], (m) [46], (n) [113], (o) [114].

Figure 6 shows the processing times reported for classifying a gesture with SoCs. Most of the processing times recorded in these studies do not show significant differences, as they are around 5 ms, highlighting the use of complex classification methods, such as neural networks. For example, in [111], a processing time of 7.89 ms is reported using CNN, while in [37], 11 ms is recorded when using an MLP. Significant variations in processing times can also be observed even when using the same method. In [46,113], MLP-based classification reports processing times of 9.75 ms and 1.61 ms, respectively. This discrepancy is mainly attributed to the model’s architecture, as in neural networks, factors such as the number of neurons, activation functions, input size, and other parameters influence processing performance. In contrast, the study of [32] stands out in the Figure for reporting the highest processing time, with 35 ms. This is due to its use of a sliding window classification process, executing 10 predictions at 3.5 ms each.

### 4.6. Neuromorphic System

Neuromorphic chips are devices whose architecture is designed to replicate the functioning of the human brain [1,130]. They enable the efficient implementation of algorithms for neural networks, as they process information similarly to the biological nervous system [12]. These chips, such as ODIN^®^ and Loihi^®^, are designed to process large amounts of data in parallel, making them ideal for applications that require high-performance signal processing, such as the classification of EMG signals when multiple sensors acquire the signals [131]. These devices are well suited for artificial intelligence tasks as they can process large amounts of data in real-time while having low energy consumption. In [132], it was reported that the DYNAP-SE^®^ chip requires only 0.05 W to classify three movements with an SNN model. Table 8 presents several studies using neuromorphic chips to classify EMG signals.

**Figure 7:**
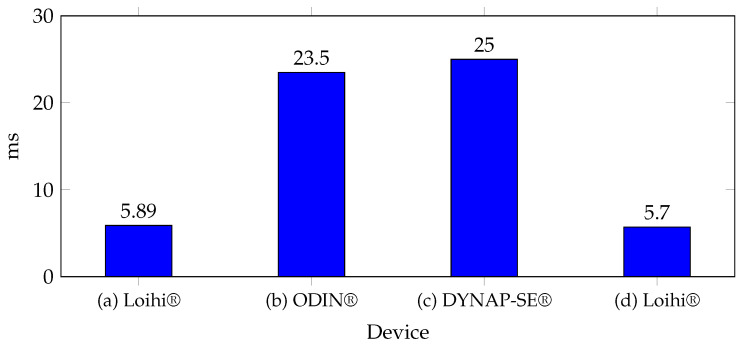
Processing times for different EMG classification techniques implemented on neuromorphic system. References: (a) [1], (b) [1], (c) [132], (d) [136].

Figure 7 shows some of the recorded results; the results of these studies demonstrate the fast response of these architectures, even when using more complex models and larger datasets in [1]—using Loihi^®^, a response time of just 5.89 ms was achieved with 96% accuracy in motion prediction. A similar time was reported in [136], where the same neuromorphic device was used, recording 5.7 ms. In [1], using ODIN^®^, and in [132], using DYNAP-SE^®^, the classification time was approximately 25 ms, which remains remarkably fast considering the low energy consumption. In [137], a processing time of 50 ms was reported; however, it was not included in the graph since this time includes classification signal acquisition and processing stages.

In general, neuromorphic devices provide a suitable processing time for real-time applications. However, architectures such as that in [88] using FPGAs and in [1] using SoCs have shown even lower processing times when using the SNN method, which is popular in neuromorphic chips. Additionally, these devices stand out for their extremely low energy consumption compared to edge computing devices.

## 5. Which Processor to Use in EMG Signal Classification?

The choice of the appropriate device for classifying EMG signals does not directly affect the classification accuracy. Instead, it influences the efficiency and complexity of the model during implementation. Robust or complex classification models require computing systems that meet the processing requirements for real-time applications. This relationship results in two approaches: Complex classification algorithms require robust hardware devices. At the same time, optimized embedded systems enable the execution of algorithms tailored to their resources in portable or everyday-use environments. The challenge for the user is, therefore, to find a balance between the complexity of the model, which affects classification accuracy, and its ability to be embedded in devices that meet the application’s requirements.

The embedded architectures analyzed in this study offer specific advantages and limitations. These depend on processing speed, energy consumption, and device costs. Table 9 summarizes each architecture’s main advantages and limitations.

It is important to note that the performance in terms of classification accuracy shows minimal differences when the same algorithm is implemented on different devices. These minor variations are usually due to implementation-specific factors, such as hardware configuration, optimization techniques, or data processing. Therefore, architectural limitations have little to no impact on classification accuracy. With this in mind, the choice of processor should focus on application-specific requirements such as the processing time required to make a decision, energy consumption, and portability rather than focusing solely on higher classification accuracy.

Table 10 presents relevant research involving different devices and their characteristics.

Edge inference is becoming increasingly popular for portable applications, emphasizing the importance of embedded devices for real-time EMG signal classification and similar applications. SoCs are characterized by their ability to execute complex artificial intelligence algorithms efficiently. Meanwhile, neuromorphic chips represent an emerging option with promising latency and energy efficiency characteristics. Ultimately, the choice of device depends on how the needs and specific application requirements are assessed. These include the required classification speed, the number of sensors used for signal acquisition, the number of movements to be identified, the robustness of the model used, and the budget available for the study. In addition, it is important to consider the level of programming or design knowledge required for the device when selecting the most suitable solution. For a project that requires a robust algorithm and a limited budget, for example, a Raspberry Pi^®^ could be the right choice. For applications that require multiple tasks at the same time, such as using multiple sensors and high processing speed, an FPGA would be a better choice.

Although the choice of device varies by application, the following guidelines can be considered based on the reviewed research:

- For low-cost and low-power applications: microcontrollers or DSPs.
- For high-speed and parallel processing: FPGAs.
- For flexibility and simple programming with complex models: SoCs.
- For applications with extremely low power consumption, high energy efficiency, and parallel processing: neuromorphic chips.

### Evolution of Devices Used in EMG Analysis and Types of Gestures

**Figure 8:**
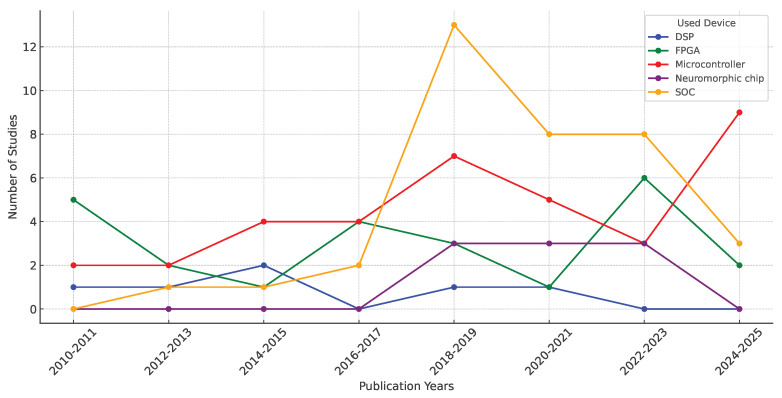
Evolution of device embedded usage in EMG studies.

The trend in the use of embedded devices for EMG classification over the years provides a global overview of the amount of literature available on this topic. This allows users to determine whether the solution they are looking for is already mature and to take advantage of previous research. Figure 8 shows a line chart illustrating the evolution of the use of embedded devices.

Figure 8 provides an overview of the use of embedded devices in EMG analysis over the years. A continuous use of microcontrollers can be observed, with a significant increase in recent years, indicating a growing demand for portable technologies. This suggests that microcontrollers are the most widely used technology due to their extensive documentation and lower cost compared to other technologies. In contrast, DSPs have only been used sporadically, and there have been times when their use has not been reported, suggesting that they are one of the least widely used technologies for this type of application. This is mainly due to the integration of DSP functions into other architectures, such as microcontrollers and FPGAs, which incorporate DSP modules into their internal structure. On the other hand, a gradual increase in FPGA usage can be observed, especially in recent years, indicating an increasing demand for parallel processing and optimization of energy consumption in embedded applications. SoCs have grown significantly since 2018 and have become the most popular technology this year. This suggests a shift in preference towards highly integrated devices where programming complex algorithms for wearable applications is more accessible, highlighting the increasing use of artificial intelligence algorithms in EMG signal processing. Meanwhile, neuromorphic chips have seen a limited number of trials, particularly between 2018 and 2023, indicating ongoing development compared to other technologies. However, their application in EMG may expand as neuromorphic architectures continue to improve.

**Figure 9:**
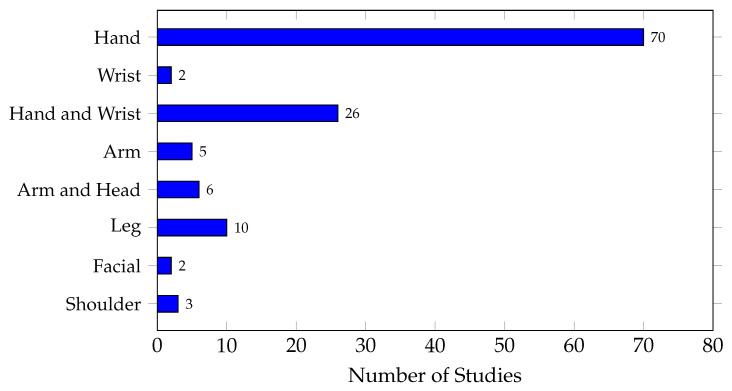
Comparison of studies analyzing each EMG gesture type.

Another important aspect to highlight is the type of gestures or movements analyzed in the reviewed studies. Figure 9 shows the distribution of EMG gesture types analyzed in embedded implementations. It can be observed that most studies focused their analysis on hand movements, with a total of 70 studies, followed by combined hand and wrist gestures with 26 studies. This suggests a clear trend in the analysis of upper limb gestures. This result is justified as the hand is the most important body part used in human–machine interfaces and in the control of prostheses and rehabilitation applications. In contrast, leg movements (10 studies), shoulder movements (3 studies), arm movements with the elbow (5 studies), and facial gestures (2 studies) were studied significantly less.

Since embedded devices rely on their processing capacity to implement complex classification procedures, the complexity of the recorded signal source can influence the classification process. Therefore, it is important to consider the type of movements analyzed when selecting a device to ensure efficient and accurate classification. Regarding comparability, gestures from different body parts, such as hand, arm, and leg, can use similar signal processing, feature extraction techniques, and classification algorithms. However, their biomechanical properties and noise distribution must be accounted for [139]. Hand and arm signals are primarily associated with fine motor tasks that require precision. EMG signals from the legs may have a higher noise level as they are influenced by external factors such as ground contact. In addition, these signals originate from a larger group of muscles than the arm, resulting in more significant variability. On the other hand, facial gestures tend to be less comparable as they originate from smaller muscles with lower-amplitude activations [139,140].

## 6. Conclusions

This article gives an overview of the implementation of EMG signal classifiers in different embedded systems. It shows that each architecture has advantages and disadvantages regarding accuracy, processing time, power consumption, and cost. Microcontrollers and DSPs are suitable for low-cost and low-power applications, while FPGAs and SoCs are ideal for tasks requiring high speed and reconfiguration flexibility. In addition, FPGAs offer parallel processing, which can be helpful for a more significant number of sensors. On the other hand, neuromorphic chips provide a promising solution for applications that require energy efficiency and real-time processing. However, as these are new technologies, the steep learning curve and the price are high. The choice of the appropriate device depends on the specific requirements of the application and the limitations of the intended use. The following recommendations can serve as a guide for developers of embedded systems working with EMG-based applications:

- Use microcontrollers or DSPs for portable and low-cost projects.
- Use FPGAs for high-speed applications with multiple inputs.
- Use SoCs for simple implementation of complex algorithms.
- Use neuromorphic chips for environments with extreme power constraints.

As a recommendation, it is suggested to avoid the use of certain architectures based on their main limitations, as outlined below:

- The computational capacity of microcontrollers is limited compared to the other analyzed devices.
- The multitasking capability of DSPs is restricted, and as algorithm complexity increases, energy consumption also rises.
- FPGAs are complex to program without a high-level compiler, and their internal resources are constrained based on cost.
- SoCs have the highest energy consumption compared to the other analyzed devices.
- The market availability of neuromorphic systems is limited, and specialized programming skills are required.

Finally, this work emphasizes the importance of understanding the necessary balance between device resources and model complexity when selecting an embedded architecture for EMG signal classification. The analysis provides a valuable reference for identifying the most suitable device for a given application, such as smart prostheses, portable medical devices, or wearable gesture control systems.

This review addresses current and emerging techniques as well as the challenges related to portability, resource optimization, and cost reduction. It contributes to the development of real-time and energy-efficient solutions in fields such as biomedical engineering and provides a guide for future research and innovation in this area.

## Figures and Tables

**Table 1:** Features used in the reviewed works.

Features	Acronym	Domain	Features	Acronym	Domain
Autoregression	AR	Time	Median power frequency	MDF	Frequency
Average threshold crossing	ATC	Time	Modified Waveform Length	MWL	Time
Cepstrum	CR	Frequency	Multiplication of Power and Peaks	MPP	Time
Centroid of frequency	CF	Frequency	Multiplication of Zeros and Peaks	MZP	Time
Continuous wavelet transform	CWT	Time–Frequency	Natural log of Variance	InVAR	Time
Dense scale-invariant feature transform	DSIFT	Time–Frequency	Number of peaks	NP	Time
Difference between moments	DBM	Time	Power spectral density	PSD	Frequency
Discrete wavelet transform	DWT	Time–Frequency	Principal Components Analysis	PCA	Time
Entropy	SE	Time	Root mean square value	RMS	Time
Fourier transform	FFT	Frequency	Root Sum Square	RSS	Time
High order statistic	HOS	Time	Signal-to-noise ratio	SNR	Frequency
Integrated absolute value	IAV	Time	Simple square integral	SSI	Time
Integrated EMG	IEMG	Time	Skewness	SK	Time
Kurtosis	K	Time	Slope changes	SSC	Time
Max value	Max	Time	Standard deviation	SD	Time
Mean absolute deviation	MAD	Time	Short Time Fourier Transform	STFT	Time–Frequency
Mean absolute value	MAV	Time	Total harmonic distortion	THD	Frequency
Mean crossing rate	MCR	Time	Variance	VAR	Time
Mean value	Mean	Time	Wavelength	WL	Time
Mean power frequency	MNF	Frequency	Wavelet transform	WT	Time–Frequency
Mean squared value	MSV	Time	Willson amplitude	WAMP	Time
Median frequency	MF	Frequency	Zero crossings	ZC	Time

**Table 2:** Feature extraction on embedded devices.

Ref.	Feature	Device	Ref.	Feature	Device
[10]	IEMG, MAV, RMS, SSI, VAR, WL	Zynq^®^ XC7Z020	[11]	MAV, VAR, WAMP, WL, ZC	Jetson Nano^®^
[16]	Mean, SD, SNR	Arduino Nano IoT^®^	[18]	DSIFT, MAV	FPGA
[19]	PCA	TMS320F28335^®^ DSP	[20]	RMS	FPGA Kintex^®^ 7
[21]	MODWT	FPGA Kintex Ultrascale^®^	[22]	RMS	FPGA Cyclone V^®^
[23]	MAD, MAV, PSD, SD, SE, SNR, THD, VAR	Arduino UNO^®^	[24]	RMS	FPGA
[25]	K, Max, Mean, Min, RMS, SD, Sk	Arduino Nano^®^	[26]	IMAV, SSC, WL, ZC	Zynq-7000^®^
[27]	CR, DWT, RMS	ARM Cortex-M4^®^ (STM32L496ZGTx)	[28]	MAV, WL, ZC	FPGA ZC706
[29]	MAV, MF, RMS, WL, ZC	Raspberry Pi^®^	[30]	MDF, MNF, RMS	Raspberry Pico^®^
[31]	RMS, SSC, WL, ZC	32-bit DSP ARM Cortex-M4^®^ core	[32]	MAV, RMS	Raspberry Pi 3^®^
[33]	DBM, MPP, MZP, MWL, NP, ZC	32-bit DSP ARM Cortex-M4^®^ core	[34]	MAV, MCR, SSC, VAR, WAMP, WFL, ZCR	ATSAML21E18B^®^ Microcontroller
[35]	MODWT	FPGA xcku035-fbva676-3-e	[36]	MAV, RMS, SSI, VAR	Raspberry Pi 3^®^
[37]	MAV, RMS, VAR, WL	Raspberry Pi 3^®^	[38]	AR, FFT, MAV, SSC, WL, WT, ZC	TMS320F28335^®^ DSP
[39]	ZC	STM32F429^®^ Microcontroller	[40]	MAV, SSC, WL, ZC	Freescale MPC5566^®^ Microcontroller
[41]	MAV, SSC, WL, ZC	Altera Stratix II^®^ FPGA			
[42]	MAV, RMS, SD, VAR	STM32f103c8t6^®^ Microcontroller	[43]	RMS	Arduino UNO^®^
[44]	MAV, MVC, SSC, WL	TMS320F2812^®^ DSP	[45]	MAV, RMS, VAR	Raspberry Pi 3 B+^®^
[46]	AR, IEMG, K, MAD, MSV, RMS, SK, VAR	Raspberry Pi 3 B+^®^	[47]	ATC	ARM Cortex-M4F^®^ Microcontroller
[48]	MAV, RMS, SD, VAR	Raspberry Pi 3^®^	[49]	HOS	FPGA Spartan-3^®^
[50]	CF, RMS, SD	PSOC Microcontroller	[51]	FFT	PSOC Microcontroller
[52]	FFT	Microcontroller	[53]	MAV, SSC, WL, ZC	Intel Atom Z530^®^
[54]	MAV	Pynq-Z1^®^ (ARM Cortex-A9^®^ & FPGA)	[55]	MAV, SSC, WL, ZC	Freescale MPC5566^®^ Microcontroller
[56]	Mean, RMS, WL	STM32F205^®^ Microcontroller	[57]	IAV, RMS	Microcontroller
[58]	AR	DSP TMS320C31^®^	[59]	AR (Order 4)	DSP TMS320C31^®^
[60]	AR, IEMG, SSC, VAR, WL, WAMP, ZC	DSP TMS320C31^®^	[61]	AR, CR, IEMG, SSC, VAR, WL, WAMP, ZC	DSP TMS320C31^®^
[62]	RMS	FPGA ALTERA DE2 demo board^®^			

**Table 3:** Classification algorithms used in the reviewed works.

Acronym	Classification Algorithm
BNN	Binarized Neural Network
BPNN	Backpropagation Neural Network
CKLM	Cascaded Kernel Learning Machine
CNN	Convolutional Neural Network
CRNN	Convolutional Recurrent Neural Network
DNN	Deep Neural Network
DT	Decision Tree
ELM	Extreme Learning Machine
FCNN	Fully-Connected Neural Network
KNN	k-Nearest Neighbors
LDA	Linear Discriminant Analysis
LSM	Liquid State Machine
LWNN	Lightweight Neural Networks
MMLD	Modified Maximum Likelihood Distance
MLP	Multilayer Perceptron
NB	Naive Bayes
PNN	Probabilistic Neural Network
QDS-CNN	Ultra-Lightweight Depth Separable Convolutional Neural Network
RBF	Radial Basis Function
RLS	Recursive Least Squares
RNN	Recurrent Neural Network
RSNN	Recurrent Spiking Neural Networks
Siamese–LSTM	Siamese Model with Long Short-Term Memory
SLR	Simple Logistic Regression
SNN	Spiking Neural Networks
SVM	Support Vector Machines
TCN	Temporal Convolutional Network

**Table 4:** Classification of EMG signals in microcontrollers.

Ref.	Device	Gestures	Accuracy %	Method
[8]	ARM Cortex M4^®^	7	90	LDA
[9]	ARM Cortex M4^®^	6	94.14	SVM
[16]	Arduino Nano IoT^®^	6	90	MLP-DT
[23]	Arduino UNO^®^	4	83.9	SVM
[25]	Arduino Nano 33 BLE Sense^®^	3	95	MLP
[27]	STM32L496ZGTx^®^	5	95.34	MLP
[34]	ATSAML21E18B (ARM Cortex-M0+)	-	99	RNN
[38]	TMS320F28335^®^	6	93.4	SLR
[42]	STM32f103c8t6^®^	2	83	NB
[47]	ARM Cortex-M4F^®^	6	96.34	FCNN
[56]	STM32F205^®^	4	-	MLP
[67]	ARM Cortex-A8 (Gumstix Overo Air^®^)	3	93.5	LDA
[68]	STM32F407ZGT6^®^	11	95	LDA
[69]	STM32F405RGT6^®^	4	-	-
[70]	Arduino Nano 33 BLE Sense^®^	-	89.4	CNN
[71]	Arduino ATmega 328p^®^	-	-	Pattern recognition
[72]	ARM Cortex-M4^®^	6	94.7	KNN
[73]	Teensy 4.0 (Cortex-M7)	-	-	Feature-based algorithms
[74]	MPC5566^®^	4	100	LDA
[75]	Arduino DUE^®^	4	97.7	MLP
[76]	ARM Cortex-M4^®^	5	92.36	SVM
[77]	Atmel SAM4S16^®^	5	92	SVM
[78]	Arduino Nano BLE 33^®^	4	87.57	DNN
[79]	GAP8 IoT^®^	8	93.7	TCN
[80]	TMS320F28069M	3	97	-

**Table 5:** Classification of EMG signals in DSP.

Ref.	Device	Gestures	Accuracy %	Method
[19]	TMS320F28^®^	3	93.5	MLP
[31]	DSP unit on ARM Cortex-M4^®^	6	94	LDA
[33]	DSP unit on ARM Cortex-M4^®^	6	92	LDA
[44]	TMS320F2812^®^	2	90	-
[58]	TMS320C31^®^	8	93.54	CKLM
[59]	TMS320C31^®^	5	95	MMLD
[61]	TMS320C31^®^	8	87.5	BPNN
[81]	dsPIC33FJ25^®^	5	91	SVM
[82]	TMS320VC5509A^®^	9	79.5	LDA
[83]	dsPIC30f4013^®^	2	-	Fuzzy logic

**Table 6:** Classification of EMG signals in FPGA and ZYNQ.

Ref.	Device	Gestures	Accuracy %	Method
[1]	FPGA	-	95.6	SNN
[10]	Zynq^®^ XC7Z020	5	98	kNN
[18]	FPGA	6	97	SVM
[20]	Kintex 7^®^ XC7K325T	17	59	SVM
[21]	Kintex Ultrascale^®^	6	95	CNN
[22]	Cyclone V^®^	9	94	KNN
[26]	Zynq-7000^®^	8	-	LDA, SVM
[28]	Zynq^®^ ZC706	6	92	NB
[35]	xcku035-fbva676-3-e^®^	2	90	Siamese network CNN
[40]	Altera Stratix III^®^	3	-	LDA
[41]	Altera Stratix III^®^	2	-	LDA
[84]	Altera Stratix III^®^	12	71.46	MLP
[86]	Pynq-Z1^®^	5	98	SVM
[87]	Intel MAX 10 10M50DAF484C7G^®^	9	95.4	BNN
[88]	Lattice iCE40UP5k^®^	12	83.17	SNN
[89]	Zynq^®^ ZC7030FBG484-3	40	90	-
[90]	Cyclone II^®^	3	87	-
[91]	PYNQ-Z1^®^	-	-	RLS
[92]	Spartan-3ADSP^®^	-	-	FFT-ANN
[93]	Spartan-3ADSP^®^	3	-	FFT analysis
[94]	Spartan 3^®^	14	91.2	ANN
[95]	Virtex-II Pro^®^	4	95.5	SVM
[96]	Altera Cyclone V^®^	-	93.97	SVM
[97]	Altera Cyclone V^®^	-	-	STFT analysis
[98]	Zynq^®^ XCZU9EG	18	98.47	QDS-CNN
[99]	Xilinx 7^®^ Series	-	-	ANN
[100]	Virtex^®^ XCV3000-4FG676	4	96.98	PNN
[101]	xcku5p-ffva676-3-e^®^	12	99.4	Siamese–LSTM
[102]	Altera Cyclone V^®^	-	94	SVM
[103]	Spartan-6^®^ XC6SLX45	17	91.5	SVM
[104]	Altera DE2-115^®^	7	85.23, 83.3	CNN, RNN
[105]	Zedboard^®^	5	63.98	SNN

**Table 7:** Classification of EMG signals in SoC.

Ref.	Device	Gestures	Accuracy %	Method
[1]	Jetson Nano^®^	-	94.8	SNN
[11]	Jetson Nano^®^	5	98	SVM
[17]	Raspberry Pi 3B+^®^	9	99	RBF
[29]	Raspberry Pi^®^	17	90.9	RVFLN
[36]	Raspberry Pi 3B+^®^	4	98.4	DT
[37]	Raspberry Pi 3B+^®^	11	96.3	MLP
[45]	Raspberry Pi 3B+^®^	4	94.06	KNN
[46]	Raspberry Pi 3B+^®^	9	99	MLP
[48]	Raspberry Pi 3^®^	7	97.39	ELM
[106]	Raspberry Pi 3B+^®^	2	96.3	DT
[107]	Jetson Nano^®^	8	98.2	CNN
[108]	Raspberry Pi 2^®^	3	90.4	LDA
[109]	Raspberry Pi 3B+^®^	6	91.66	CNN
[110]	Jetson Nano^®^	10	96	CRNN
[111]	Jetson TX2^®^	14	84.2	CNN
[112]	Jetson TX2^®^	15	91.26	CNN
[113]	Raspberry Pi 3B+^®^	8	92	MLP
[114]	SoC PULP^®^	3	88	SVM
[115]	Raspberry Pi 3^®^	8	99.9	SVM
[116]	Raspberry Pi 3^®^	2	93.3	SVM
[117]	Raspberry Pi^®^	5	93.5	NB
[118]	Raspberry Pi^®^	10	78	SVM
[119]	Raspberry Pi 3^®^	5	86.39, 73.61	SVM, KNN
[120]	Raspberry Pi 3^®^	10	85.53	ELM
[121]	Raspberry Pi 3B+^®^	2	80, 70, 50	KNN, SVM, LDA
[122]	Raspberry Pi 3B+^®^	-	90.3	SVM
[123]	Raspberry Pi^®^	-	94.23	CNN
[124]	Raspberry Pi^®^	2	92.35	CNN
[125]	Jetson Nano^®^	5	92.5	SVM
[126]	Jetson Nano^®^	-	95	RNN
[127]	Jetson Nano^®^	8	-	CNN
[128]	Jetson Nano^®^	8	88.54	CNN
[129]	Jetson Nano^®^	21	82.93	CNN

**Table 8:** Classification of EMG signals in neuromorphic system.

Ref.	Device	Gestures	Accuracy %	Method
[1]	Intel Loihi^®^	-	96	SNN
[1]	ODIN^®^ + MorphIC^®^	-	89.4	SNN
[12]	Intel Loihi^®^ (Nahuku 32)	3	90	RSNN
[130]	DYNAP-SE^®^	-	77, 73.3	SRNN, SVM
[131]	Intel Loihi^®^	-	92.2	SNN
[131]	ODIN^®^ + MorphIC^®^	-	85.1	SNN
[132]	DYNAP-SE^®^	3	84, 81	SNN, SVM
[133]	DYNAP-SE^®^	2	-	LSM
[134]	SpiNNaker^®^	4	84.4	SNN
[135]	DYNAP-SE^®^	3	55.92	SRNN
[136]	Intel Loihi^®^ (Kapoho Bay)	12	74	SNN
[136]	Intel Loihi^®^ (Nahuku 32 board)	3	90	SRNN

**Table 9:** Advantages and limitations of different architectures for EMG signal classification.

Architecture	Advantages	Limitations
Microcontroller	- Low cost and widely available compared to other architectures reviewed. - Energy efficient, ideal for portable devices [27]. - Multi-sensor support for EMG signal acquisition [69].	- Limited processing power, not suitable for complex algorithms [8]. - They are not ideal for high data bandwidth [138].
DSPs	- Optimized for real-time signal processing [82]. - Moderate energy consumption compared to other architectures reviewed.	- More complex algorithms require higher energy consumption [58]. - Limited capacity for handling multiple tasks or complex algorithms [19].
FPGAs	- High level of customization and parallelism for complex algorithms [104]. - Real-time processing with low latency [94]. - Suitable for handling large datasets efficiently [104].	- Limited scalability [102]. - Requires expertise in hardware programming (e.g., Verilog or VHDL) [104].
SoCs	- Combines processing, storage, and connectivity in a single chip. - Supports high-level programming languages like Python [111,113]. - Balanced performance between computational power and energy consumption [113].	- Higher energy consumption compared to microcontrollers. - Lower latency than solutions with FPGAs or DSPs [46,113].
Neuromorphic Chips	- Exceptionally energy efficient, mimicking brain function [136,137]. - High parallel processing capacity for real-time applications [136]. - Compact size for portable systems [137].	- Limited availability in the market. - High initial cost and requires specialized programming skills [137].

**Table 10:** Summary of the main works analyzed.

Ref.	Device	Method	Gestures	Sensors	Processing Time (ms)	Power (W)	Accuracy (%)	Price 2025 USD
[9]	ARM Cortex M4^®^	SVM	7	8	-	0.029	90	[5–30]
[31]	DSP not specific	LDA	6	8	75	0.04	94	[20–100]
[26]	FPGA Zynq-7000^®^	SVM	8	192	15	-	97	[100–1800]
[86]	FPGA Pynq-Z1^®^	SVM	5	8	0.015	3.1	98	[300–500]
[107]	Jetson Nano^®^	CNN	8	32	5	3.055	98.2	[60–250]
[29]	Raspberry Pi^®^	SVM	17	12	10.5	4	90.4	[35–270]
[17]	Raspberry Pi 3B+^®^	RBF	9	8	4.5	4	99.03	[40–100]
[132]	DYNAP-SE^®^	SNN	3	8	25	0.05	84	Manufacturer quotes available upon request
[1]	Intel Loihi^®^	SNN	-	-	5.89	0.03	96	Manufacturer quotes available upon request

## Data Availability

Not applicable.

## Appendix A. Device Manufacturers

This appendix shows the manufacturers, cities and countries of the devices named in this work, which are listed in Table A1.

**Table A1:** Device manufacturers mentioned in the article.

Device	Manufacturer	City	Country
Arduino Nano 33 BLE Sense^®^	Arduino^®^	Ivrea	Italy
Arduino Nano^®^	Arduino^®^	Ivrea	Italy
Arduino UNO^®^	Arduino^®^	Ivrea	Italy
ATSAML21E18B^®^ Microcontroller	Microchip^®^	Chandler	USA
DSP dsPIC33FJ25^®^	Microchip^®^	Chandler	USA
DSP TMS320VC5509A^®^	Texas Instruments^®^	Dallas	USA
FPGA Cyclone V^®^	Intel^®^ (Altera^®^)	San José	USA
FPGA Kintex 7^®^	Xilinx^®^	San José	USA
FPGA MAX 10^®^	Intel^®^	Santa Clara	USA
FPGA Stratix III^®^	Intel^®^ (Altera^®^)	San José	USA
Freescale MPC5566^®^	Freescale^®^ Semiconductor	Austin	USA
Intel Atom Z530^®^	Intel^®^	Santa Clara	USA
Intel Loihi^®^	Intel^®^	Santa Clara	USA
Jetson Nano^®^	NVIDIA^®^	Santa Clara	USA
Jetson TX2^®^	NVIDIA^®^	Santa Clara	USA
Pynq-Z1^®^	Xilinx^®^	San José	USA
Raspberry Pi 3^®^	Raspberry Pi Ltd^®^	Cambridge	United Kingdom
Raspberry Pi 3B+^®^	Raspberry Pi Ltd^®^	Cambridge	United Kingdom
SoC PULP^®^	ETH Zurich^®^	Zürich	Switzerland
Spartan-3^®^ FPGA	Xilinx^®^	San José	USA
STM32F429^®^ Microcontroller	STMicroelectronics^®^	Geneva	Switzerland
TMS320F28335^®^ DSP	Texas Instruments^®^	Dallas	USA
Zynq-7000^®^ FPGA	Xilinx^®^	San José	USA
